# Fit for genomic and proteomic purposes: Sampling the fitness of nucleic acid and protein derivatives from formalin fixed paraffin embedded tissue

**DOI:** 10.1371/journal.pone.0181756

**Published:** 2017-07-25

**Authors:** Anna Yakovleva, Jordan L. Plieskatt, Sarah Jensen, Razan Humeida, Jonathan Lang, Guangzhao Li, Paige Bracci, Sylvia Silver, Jeffrey Michael Bethony

**Affiliations:** 1 Department of Microbiology, Immunology and Tropical Medicine, The George Washington University, Washington DC, United States of America; 2 AIDS and Cancer Specimen Resource (ACSR), The George Washington University, Washington DC, United States of America; 3 Department of Epidemiology and Biostatistics, University of California, San Francisco, San Francisco, CA, United States of America; 4 AIDS and Cancer Specimen Resource (ACSR), University of California at San Francisco, San Francisco, CA, United States of America; University of Nottingham, UNITED KINGDOM

## Abstract

The demand for nucleic acid and protein derivatives from formalin-fixed paraffin-embedded (FFPE) tissue has greatly increased due to advances in extraction and purification methods, making these derivatives available for numerous genomic and proteomic platforms. Previously, DNA, RNA, microRNA (miRNA), or protein derived from FFPE tissue blocks were considered “unfit” for such platforms, as the process of tissue immobilization by FFPE resulted in cross-linked, fragmented, and chemically modified macromolecules. We conducted a systematic examination of nucleic acids and proteins co-extracted from 118 FFPE blocks sampled from the AIDS and Cancer Specimen Resource (ACSR) at The George Washington University after stratification by storage duration and the three most common tumor tissue types at the ACSR (adenocarcinoma, squamous cell carcinoma, and papillary carcinoma). DNA, RNA, miRNA, and protein could be co-extracted from 98% of the FFPE blocks sampled, with DNA and miRNA “fit” for diverse genomic purposes including sequencing. While RNA was the most labile of the FFPE derivatives, especially when assessed by RNA integrity number (RIN), it was still “fit” for genomic methods that use smaller sequence lengths, *e*.*g*., quantitative PCR. While more than half of the protein derivatives were fit for proteomic purposes, our analyses indicated a significant interaction effect on the absorbance values for proteins derived from FFPE, implying that storage duration may affect protein derivatives differently by tumor tissue type. The mean absorbance value for proteins derived from more recently stored FFPE was greater than protein derived from older FFPE, with the exception of adenocarcinoma tissue. Finally, the fitness of one type of derivative was weakly associated with the fitness of derivatives co-extracted from the same FFPE block. The current study used several novel quality assurance approaches and metrics to show that archival FFPE tissue blocks are a valuable resource for contemporary genomic and proteomic platforms.

## Introduction

The demand for high-quality molecular derivatives from archived formalin-fixed paraffin-embedded (FFPE) tissue from biorepositories has greatly increased in recent years due to the low cost, high-throughput, and accessibility of contemporary genomic and proteomic platforms [1, 2]. This is despite the fact that FFPE blocks are often considered a poor source for genomic and proteomic material. As with many biobanked samples [3], the requirements of FFPE for use in contemporary research platforms were not known when the tumor tissues were originally fixed, a particularly critical issue for nucleic acids and proteins, which are tightly cross-linked into the FFPE matrix. While FFPE immobilization preserves tissue morphology, including cytological details and the immunoreactivity of tissue antigens, this crosslinking has deleterious effects on the embedded nucleic acids and proteins, such as chemical modifications, degradation, and even sequence alterations [4]. However, recent advances in the extraction and purification of nucleic acids and proteins from FFPE blocks have now made these derivatives highly accessible for genomic and proteomic analyses [5–8], though the “quality” or the “fitness” of these derivatives remains unpredictable. Herein, a fit-for-purpose approach was used to assess the "fitness" of DNA, RNA, miRNA, and protein derivatives co-extracted from FFPE blocks archived in the AIDS and Cancer Specimen Resource (ACSR) at The George Washington University (GWU). The objective of this Fit For Purpose (FFP) study was to provide biobanks with a framework to apply in similar studies to assess nucleic acids and protein derivatives from FFPE tissue blocks [3, 9] and to produce evidence for the critical role of biorepositories as partners in cancer research.

## Materials and methods

### Study site

This study was done under the approval of the George Washington University Institutional Review Board (IRB#069498). FFPE blocks were sampled from the ACSR at GWU, where they were acquired from diverse geographical locations from 1990 to 2013 and stored at 21–23°C in a temperature monitored room. The ACSR database, which includes data for the storage location and the annotation of the FFPE blocks, was used to generate the stratified random sample (described in detail below).

### Power and sample size calculations

A sample size of 120 FFPE cases was determined by several logistical factors, including labor, reagents, and availability of adenocarcinoma, papillary carcinoma, and squamous cell carcinoma blocks (the three most common tumor types in FFPE) in the ACSR at GWU. Statistical computation showed this sample size was sufficient to detect a twofold difference in the “fitness” between FFPE blocks from cases stored for less than 11 years (2002–2013, n = 60 cases) and greater than 11 years (1990–2001, n = 60 cases) assuming an alpha = 0.05 and a beta = 0.20.

### Block inclusion criteria, stratification, and randomization

The ACCESS database of the ACSR at GWU was used to search for FFPE blocks by cancer case to avoid selecting multiple FFPE blocks from the same individual. A sampling frame generated from listing available FFPE blocks was stratified by storage duration (11 year intervals starting at 1990) and then by the three most common tumor tissue types in the ACSR at GWU. Simple random sampling without replacement was conducted within each of the six strata (three tissue types x two time intervals). If the first FFPE block from a sampled case did not meet the inclusion criteria described below, the next available FFPE block from the same case was sampled. If a case did not have any blocks meeting the inclusion criteria, the next case from the randomized list was selected until the sample size of 20 FFPE blocks for each storage interval and each tumor tissue type was obtained. An FFPE block had to have sufficient material to generate four 10 μm sections, with the available tissue occupying an area of at least 4 mm^2^ to be included in the study.

### Sectioning

**Fig 1:**
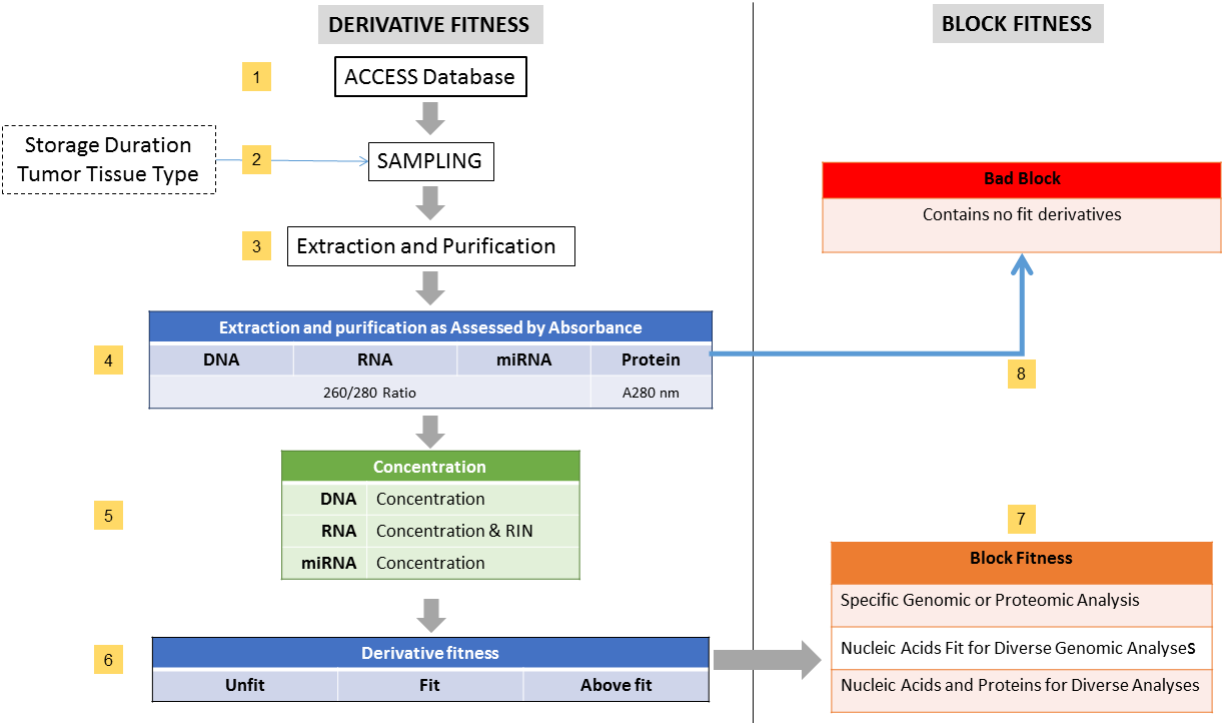
Fit for genomic and proteomic purposes by steps (numbered yellow boxes). **In step 1,** the ACCESS database of the ACSR at GWU was used to construct a sampling frame of available FFPE blocks by cancer case, which were the sampling units used to avoid selecting multiple FFPE blocks from the same individual. **In step 2,** the sampling frame of FFPE blocks was stratified by intervals of 11 years of storage (1990–2001 and 2002–2013, inclusive) and then the three tumor tissue types with the highest frequency of FFPE in the ACSR at GWU. Simple random sampling without replacement was conducted in each stratum until the targeted sample size of 20 FFPE blocks per storage duration and tumor tissue type was reached. **In step 3**, commercial kits were employed to extract nucleic acids and protein from 10 μm FFPE sections from each block; a separate FFPE section was used for each type of nucleic acid or protein extraction. **In step 4,** an initial assessment for the presence of the nucleic acid or protein was conducted by ultraviolet absorbance (UV). **In step 5,** the purity and concentration of nucleic acid and protein extracts were determined by a SpectraDrop Micro-Volume Microplate with a SPECTRAmax 384PLUS plate reader and SoftMax Pro v6.4.1 software for device control and data analysis. **In step 6,** nucleic acid and protein derivatives were assigned to fitness categories as described in the Methods section. **In step 7**, the fitness of each FFPE block was assessed by ranking the combined fitness of the derivatives as follows: FFPE blocks that met the “Fit Nucleic Acids and Proteins for Diverse Analyses” requirements included blocks in which all four derivatives (DNA, RNA, miRNA, and protein) were “Fit”. FFPE blocks that were categorized as “Fit Nucleic Acids for Diverse Genomic Analyses” included blocks that were determined to have “Fit” nucleic acid derivatives only. FFPE blocks that had one or two "fit" or "above fit" derivative out of the three nucleic acid derivatives and “unfit”, "fit" or "above fit" protein derivatives, were considered “Fit for a Specific Genomic or Proteomic Analysis”. **In step 8,** if an FFPE block had no “Fit” molecular derivatives, it was considered a “Bad Block”.

Each FFPE block was faced off and trimmed on a Microm HM315 microtome (Walldorf, Germany), with the first two 5 μm sections discarded before a 10 μm section was cut for use in the study. Each 10 μm section was collected into separate sterile 1.5 mL Eppendorf tubes and stored in a cryogenic box at −20°C. Of the FFPE blocks sampled, 120 had adequate tumor tissue by visual examination for inclusion into the study; however, after nucleic acid and protein extraction and purification only 118 (98%) of the 120 blocks had tissue that could be analyzed for absorbance and concentration as shown in Fig 1.

### Extraction and purification of nucleic acids and proteins

Standardized approaches and kits were used for nucleic acid extraction with a single 10 μm FFPE section used for each extraction. Specifically, genomic DNA was extracted from a single 10 μm FFPE section according to manufacturer’s instructions using the QIAamp® DNA FFPE Tissue kit (Qiagen, Valencia, CA) and eluted into a total volume of 50 microliters of ATE (supplied) buffer. A single 10 μm FFPE section was used to extract both miRNA using the miRNeasy FFPE kit (Qiagen, Valencia, CA) and RNA using the RNeasy® FFPE kit (Qiagen, Valencia, CA) according to manufacturer instructions and both derivatives individually eluted to 16 microliters of RNase-free water. A third 10 μm FFPE section was used to extract protein using the Qproteome® FFPE Tissue kit (Qiagen, Valencia, CA) according to manufacturer’s instructions. All samples were maintained on ice prior to absorbance and concentration determination as described below. After analysis, the samples were stored at –70°C. For protein, an additional protocol was used, in which after the initial assessment of concentration by UV absorbance (A_280_), the protein preparation was further purified according to the manufacturer’s protocol “Extraction of Proteins from FFPE Tissues and Cleanup for 2D-PAGE or MS analysis.” This involved continuing with a methanol precipitation step and eventual dissolution of the protein pellet in 20 microliters of PBS. Commercially available kits for each derivative type were used [10].

### Analysis of nucleic acids and protein extracts

All nucleic acid and protein preparations were analyzed using a SpectraDrop Micro-Volume Microplate (Molecular Devices, Sunnyvale, CA) with a SPECTRAmax 384PLUS plate reader (Molecular Devices, Sunnyvale, CA) and SoftMax Pro v6.4.1 (Molecular Devices, Sunnyvale, CA) software for device control and data analysis. Briefly, four microliters of each sample were placed on the SpectraDrop Microplate and 0.5 mm path length cover slide. Absorbance measurements were taken at 230 nm, 260 nm, and 280 nm for all samples and Spectrum wavescans stored (220 nm-350 nm with a step size of 4 nm). Absorbance ratios were determined via SoftMax Pro v6.4.1 software and concentration was determined using default software formulas with the following concentration factors: RNA (40); miRNA (40); and DNA (45). Given the presence of a heterogeneous protein preparation without known extinction coefficients, the absorbance value at 280 nm was used as the only indicator of protein concentration. RNA was also analyzed with a 2100 BioAnalyzer (Agilent Technologies, Santa Clara, CA) for RNA integrity (RIN) using the RNA 6000 Nano Kit (Agilent Technologies, Santa Clara, CA) and 2100 Expert Software. DNA was subsequently analyzed on a 2200 TapeStation (Agilent Technologies, Santa Clara, CA), utilizing the Genomic DNA ScreenTape (Agilent Technologies, Santa Clara, CA) for quality control analysis of DNA samples with the Agilent 2200 TapeStation Software. All DNA samples (one microliter) were analyzed according to manufacturer’s instructions. Results and data obtained were not included in statistical analysis but provided in S1 File.

### Ordinal fitness classification

Nucleic acid and protein derivatives were assigned to fitness categories as defined in previous publications [10, 11]. The SAS coding to convert continuous variables into mutually exclusive categorical variables can be found in S2 File. Please note that the cutoff values for fitness classification are set at two decimal places of accuracy. For the nucleic acids (DNA, RNA and miRNA), the categories of “unfit”, “fit”, and “above fit” by the absorbance ratio of A_260/280_ ratios are as follows: an A_260/280_ ratios < 1.50 or > 2.50 were considered ‘unfit”; an A_260/280_ ratio between 1.50–1.80 (inclusive of these values) and between 2.20–2.50 (inclusive of these values) were considered “fit”; and an A_260/280_ ratio ranging from 1.80–2.20 (exclusive of these values) were considered “above fit” [12] [13]. For proteins, absorbance values obtained at A_280_ were used for classification. The classifications of “unfit,” “fit” or “above fit” were based upon the expected total protein recovery from a single FFPE section. Protein values were defined as “unfit” when the A_280_ was < 1.25; “fit” when A_280_ values ranged from 1.25–2.50 (inclusive of these A_280_ values); and “above fit” when the protein values > 2.50 A_280_. The concentrations of DNA, RNA, and miRNA were divided into three categories: < 5.00 ng/μL, between 5.00–50.00 ng/μL (inclusive of these concentration values), and > 50.00 ng/μL. While various assays and applications may have sample concentration requirements, the concentration intervals presented above represent sufficient molecular extract for common genomic applications (e.g. PCR, microarray, or next generation sequencing). Ordinal assignment for RNA by RIN value was based on the published literature (see [10, 11]) as follows: RIN “unfit” was < 2.00 RIN; “fit” was a RIN within the range of 2.00–3.00 (inclusive of these RIN values); and “above fit” was a RIN > 3.00.

### Statistical analysis

Two replicate values were taken for the absorbance and concentration of DNA, RNA, miRNA and protein derivatives from each FFPE block, with the mean of the duplicates used for statistical analyses when both values were positive. In the case when duplicate readings had conflicting values, i.e., one absorbance or concentration had a value that was positive and a value that was negative, the positive value was retained for statistical analyses. In three cases, both duplicate concentration values were negative for a derivative and as such zero was assigned to their corresponding concentration measurements, implying that the sample was “unfit” for that particular derivative. An ordinal categorical classification of overall block fitness is summarized in Table 1.

**Table 1:** Classification of FFPE block fitness as determined by the quality of nucleic acid and proteins derivatives.

Fitness	Definition
Fit Nucleic Acids and Proteins for Diverse Analyses	Contains “fit” or “above fit” nucleic acids and protein derivatives in a concentration sufficient for downstream applications
Nucleic Acids Fit for Diverse Genomic Analyses	Contains a "fit" or "above fit" nucleic acid derivatives (e.g., DNA, RNA and miRNA) but "unfit" protein derivatives
Fit for a Specific Genomic or Proteomic Analysis	Only one or two "fit" or "above fit" derivative out of the three nucleic acid derivatives and “unfit”, "fit" or "above fit" protein derivatives
Bad Block (absent of any fit derivatives)	Contains nucleic acid or protein derivatives determined to be "unfit"

**Fig 2:**
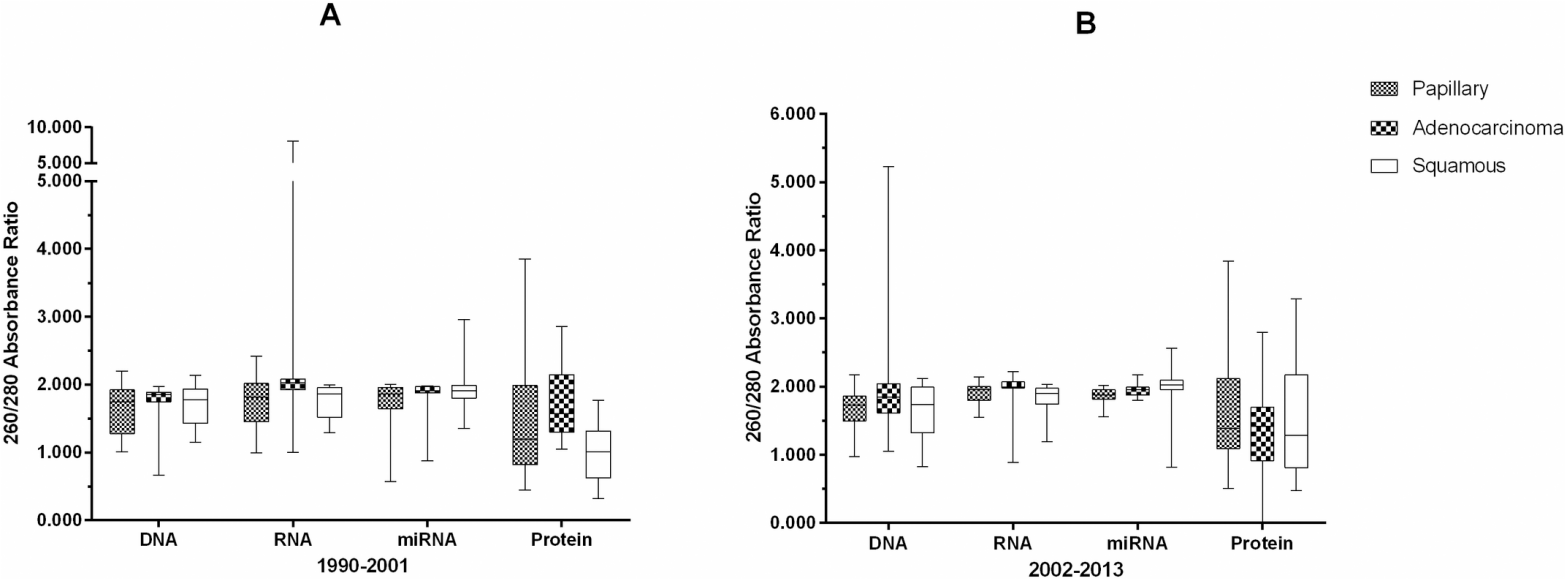
Box and whiskers plots showing the distribution of absorbance ratios (A_260/280_) for nucleic acids DNA, RNA, miRNA and for protein (A_280_) from FFPE. Panel A shows derivatives from FFPE stored between 1990–2001 and Panel B derivative from FFPE stored between 2002–2013 stratified by storage duration (11 year intervals) and cancer tumor tissue type (adenocarcinoma, squamous cell, and papillary carcinoma).

**Fig 3:**
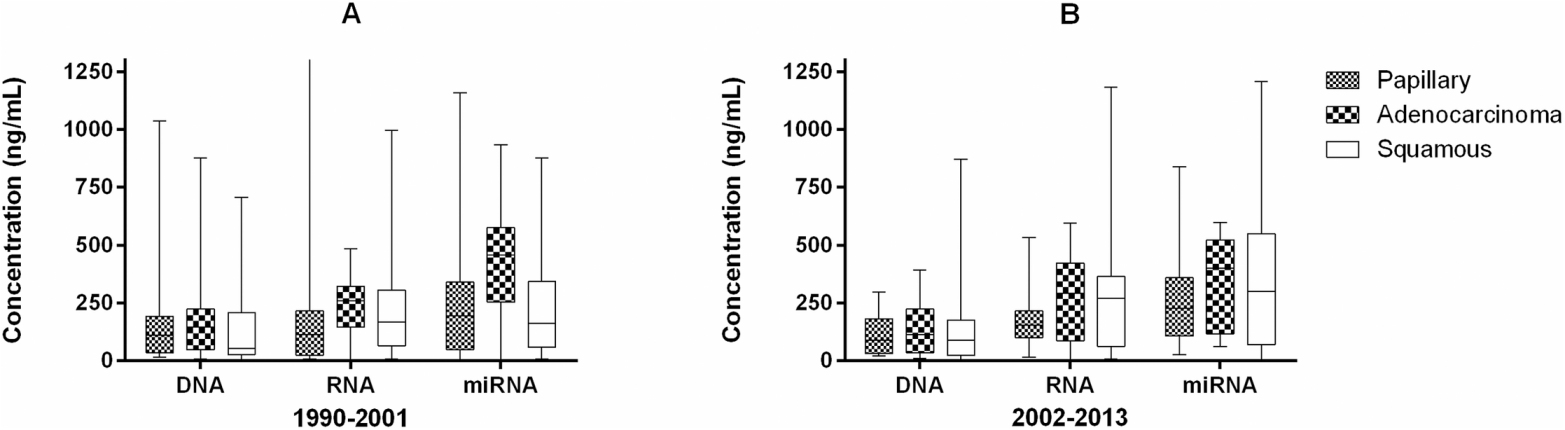
Box and whiskers plots showing the distribution of concentration (nanograms per microliter) for the nucleic acids DNA, RNA, and miRNA co-extracted from FFPE. Panel A shows derivatives from FFPE stored between 1990–2001 and Panel B shows derivative from FFPE stored between 2002–2013.

Box and whiskers plots (Figs 2 and 3) were used to depict the effect of storage duration in 11-year intervals and cancer tumor tissue type on the absorbance and concentration of derivatives derived from FFPE. A two-way analysis of variance (ANOVA) tested the effects of the two factors on these derivatives, followed by multiple pairwise comparisons with a Bonferroni correction when an overall difference among the derivatives was determined to be significant by the ANOVA. Finally, a three-way ANOVA was used to assess differences in absorbance and concentration from FFPE derivative types by cancer tumor tissue type, storage duration by 11-year intervals, and the interactions of these two factors.

In order to statistically assess the fitness of derivatives, Cochran-Mantel-Haenszel (CMH) tests were performed based on the contingency tables shown in Tables 2–5 for each derivative type. These tables evaluated the association between fitness category (i.e. “unfit”, “fit” and “above fit”) and storage duration or fitness category and cancer tumor tissue type. Besides analyzing the effects of storage duration and cancer tumor tissue type on the fitness of a derivative type, the overall fitness of each FFPE block, i.e. the combined fitness of the four derivatives co-extracted from the same FFPE block, was investigated using CMH tests. A Spearman rank correlation was used to determine the association between the grouped fitness of derivatives co-extracted from the same FFPE block. The continuous measurements of absorbance and concentration were examined as log-transformed values, since the raw data deviated from a normal distribution. Tests were considered statistically significant of *P* ≤ 0.05. Statistical analyses were conducted using SAS software, version 9.3 (SAS Institute Inc., Cary, North Carolina).

**Table 2:** The quality of DNA extracted from formalin-fixed paraffin-embedded (FFPE) tissue by the duration of storage (11 year intervals) and three tumor tissue types (n = 118).

		1990–2001						2002–2013				
				DNA 260/280 (Absorbance Ratio)								
	Unfit^1^		Fit^2^		Above Fit^3^		Unfit		Fit		Above Fit	
Tissue Type	n	%	n	%	n	%	n	%	n	%	n	%
Papillary	7	35	4	20	9	45	7	35	6	30	7	35
Adenocarcinoma	3	15	5	25	12	60	5	28	5	28	8	44
Squamous	6	30	5	25	9	45	7	35	4	20	9	45
Total	16	27	14	23	30	50	19	33	15	26	24	41
			DNA Concentration (Nanograms per microliter)									
	< 5.00		5.00–50.00		> 50.00		< 5.00		5.00–50.00		> 50.00	
Tissue Type	n	%	n	%	n	%	n	%	n	%	n	%
Papillary	0	0	8	40	12	60	0	0	7	35	13	65
Adenocarcinoma	0	0	5	25	15	75	0	0	6	33	12	67
Squamous	1	5	8	40	11	55	1	5	6	30	13	65
Total	1	2	21	35	38	63	1	2	19	33	38	66

^1^ “Unfit” refers to DNA with a A_260/280_ ratio < 1.50 or > 2.50

^2^ “Fit” refers to DNA with an A_260/280_ ratio 1.50–1.80 (inclusive of the ratio values) or an A_260/280_ ratio between 2.20–2.50 (inclusive of the ratio values)

^3^ “Above Fit” refers to DNA with an A_260/280_ ratio between 1.80–2.20 (exclusive of the ratio values).

**Table 3:** The quality of RNA extracted from FFPE by duration of storage and tumor tissue types (n = 118).

	1990–2001						2002–2013					
	RNA 260/280 (Absorbance Ratio)											
	Unfit^1^		Fit^2^		Above Fit^3^		Unfit		Fit		Above Fit	
Tissue Type	*n*	%	*n*	%	*n*	%	*n*	%	*n*	%	*n*	%
Papillary	6	30	5	25	9	45	0	0	5	25	15	75
Adenocarcinoma	3	15	0	0	17	85	1	6	2	11	15	83
Squamous	3	15	5	25	12	60	3	15	3	15	14	70
Total	12	20	10	17	38	63	4	7	10	17	44	76
	RNA Concentration (Nanograms per microliter)											
	< 5.00		5.00–50.00		> 50.00		< 5.00		5.00–50.00		> 50.00	
Tissue Type	*n*	%	*n*	%	*n*	%	*n*	%	*n*	%	*n*	%
Papillary	0	0	8	40	12	60	0	0	2	10	18	90
Adenocarcinoma	1	5	2	10	17	85	1	6	2	11	15	83
Squamous	0	0	3	15	17	85	0	0	3	15	17	85
Total	1	2	13	22	46	77	1	2	7	12	50	86
	RNA Integrity Number (RIN)											
	Unfit^4^		Fit^5^		Above Fit^6^		Unfit		Fit		Above Fit	
Tissue Type	*n*	%	*n*	%	*n*	%	*n*	%	*n*	%	*n*	%
Papillary	9	45	11	55	0	0	3	15	17	85	0	0
Adenocarcinoma	2	10	18	90	0	0	1	6	17	94	0	0
Squamous	6	30	13	65	1	5	4	20	16	80	0	0
Total	17	28	42	70	1	2	8	14	50	86	0	0

^1^ “Unfit” refers to RNA with a A_260/280_ ratio < 1.50 and > 2.50

^2^ “Fit” refers to RNA with an A_260/280_ ratio 1.50–1.80 (inclusive) or an A_260/280_ ratio between 2.20–2.50 (inclusive)

^3^ “Above Fit” refers to RNA with an A_260/280_ ratio between 1.80–2.20 (exclusive of the ratio values).

^4^ “Unfit” refers to RNA with a RIN < 2.00

^5^ “Fit” refers to RNA with a RIN within the range of 2.00–3.00 (inclusive of these RIN values); and

^6^ “Above fit” refers to RNA with a RIN > 3.00.

**Table 4:** The quality of miRNA extracted from formalin-fixed paraffin-embedded (FFPE) tissue by duration of storage (11 year intervals) and three tumor tissue types (n = 118).

	1990–2001						2002–2013					
			miRNA 260/280 (Absorbance Ratio)									
	Unfit^1^		Fit^2^		Above Fit^3^		Unfit		Fit		Above	Fit
Tissue Type	n	%	n	%	n	%	n	%	n	%	n	%
Papillary	3	15	4	20	13	65	0	0	4	20	16	80
Adenocarcinoma	2	10	1	5	17	85	1	6	1	6	16	89
Squamous	4	20	1	5	15	75	2	10	5	25	13	65
Total	9	15	6	10	45	75	3	5	10	17	45	78
			miRNA Concentration(Nanograms per microliter)									
Tissue Type	< 5.00		5.00–50.00		> 50.00		< 5.00		5.00–50.00		> 50.00	
	n	%	n	%	n	%	n	%	n	%	n	%
Papillary	1	5	5	25	14	70	0	0	1	5	19	95
Adenocarcinoma	1	5	1	5	18	90	1	6	0	0	17	94
Squamous	0	0	4	20	16	80	1	5	2	10	17	85
Total	2	3	10	17	48	80	2	3	3	5	53	91

^1^ “Unfit” refers to miRNA with a A_260/280_ ratio < 1.50 or > 2.50

^2^ “Fit” refers to miRNA with an A_260/280_ ratio 1.50–1.80 (inclusive) or an A_260/280_ ratio between 2.20–2.50 (inclusive)

^3^ “Above Fit” refers to miRNA with an A_260/280_ ratio between 1.80–2.20 (exclusive of the ratio values).

**Table 5:** The quality of protein extracted from formalin-fixed paraffin-embedded (FFPE) tissue stratified by the storage duration (11 year intervals) and three tumor tissue types (n = 118).

	1990–2001						2002–2013						
	Protein (Absorbance at 280 nm)												
	Unfit^1^		Fit^2^		Above Fit^3^			Unfit		Fit		Above Fit	
Tissue Type	*n*	%	*n*	%	*n*	%		*n*	%	*n*	%	*n*	%
Papillary	11	55	7	35	2	10		6	30	10	50	4	20
Adenocarcinoma	3	15	14	70	3	15		7	39	9	50	2	11
Squamous	14	70	6	30	0	0		10	50	7	35	3	15
Total	28	47	27	45	5	8		23	40	26	45	9	16

^1^ “Unfit” refers to protein with an A_280_ < 1.25

^2^ “Fit” refers to protein with an A_280_ 1.25–2.50 (inclusive of these values)

^3^ “Above Fit” refers to protein with an A_280_ between > 2.50.

## Results

### Descriptive statistics

#### DNA from FFPE

No significant differences in mean absorbance value ratios (A_260_/_280_) were observed for DNA, when derived from FFPE stratified by either storage duration or cancer tumor tissue type (Fig 2A and 2B). No significant differences in mean concentration of DNA were observed when stratified by storage duration or cancer tumor tissue type based on a two-way ANOVA (Fig 3A and 3B). Further interrogation of DNA samples were made with the genomic DNA TapeStation and the sizing range (base pair) along with peak (base pair size) of the DNA present in samples is provided in S1 File. This information may be considered supplemental as it is not included in the statistical model used for the Fit for Purpose study.

#### RNA from FFPE

A significant difference was observed in the mean absorbance values (A_260_/_280_) for RNA derived from FFPE, when stratified by cancer tumor tissue type when analyzed by two-way ANOVA (*P < 0.04), although no significant differences remained after Bonferroni correction for three tests (Fig 2A and 2B). No significant effect of cancer tumor tissue type or storage duration was observed on the mean concentration of RNA extracted from FFPE when assessed by two-way ANOVA (Fig 3A and 3B).

#### miRNA from FFPE

A significant difference in mean absorbance ratio (A_260_/_280_) was observed for miRNA derived from FFPE by storage duration in 11 year intervals as determined by two-way ANOVA (*P < 0.03) (Fig 2A and 2B). Subsequent multiple pairwise testing, with a Bonferroni correction, showed that the miRNA derived from recently stored FFPE (2002–2013) had an mean absorbance (A_260_/_280_) ratio 1.082 times higher than miRNA derived from older FFPE (1990–2001). No significant difference in the mean concentration of miRNA by either cancer tumor tissue type or storage duration as assessed by two-way ANOVA (Fig 3A and 3B).

#### Protein from FFPE

No significant differences were observed for the mean absorbance values (A_280_) for protein derived from FFPE when stratified by cancer tumor tissue type and storage duration as determined by two-way ANOVA (Fig 2A and 2B).

#### Comparison of DNA, RNA, miRNA and protein co-extracted from FFPE blocks

There were significant differences in mean absorbance values among the four FFPE derivatives when tested by three-way ANOVA (*P < 0.0001) consisting of (1) derivative type, (2) storage duration and (3) cancer type. The absorbance levels of proteins derived from FFPE were much lower than the absorbance levels of the three nucleic acids derived from FFPE. The mean absorbance levels of DNA, RNA and miRNA were 1.34, 1.48 and 1.49 significantly higher than the mean absorbance level of protein, respectively. A significant difference in the mean concentration of nucleic acid derivatives (DNA, RNA, miRNA) was also observed by three-way ANOVA, with the mean concentration levels of DNA derived from FFPE significantly lower (*P < 0.0001 even after Bonferroni correction) compared to the mean concentrations of RNA and miRNA derived from FFPE. The mean concentration levels of RNA and miRNA were 1.67 and 2.20 times higher than DNA.

#### Interaction analyses

The potential for the interaction of storage duration of FFPE and the tumor tissue type on absorbance and concentration values of DNA, RNA, miRNA and protein derivatives from FFPE was assessed by two-way ANOVA. No significant interaction between storage duration and cancer tumor tissue type on the mean absorbance ratio (A_260/280_) of DNA, RNA and miRNA were observed. A significant interaction by storage duration and cancer tumor type on the mean absorbance value (A_280_) of protein was observed (*P = 0.01) by two-way ANOVA. The mean absorbance value (A_280_) for proteins derived from more recently stored FFPE (2002–2013) was greater than protein derived from older FFPE (1990–2001), with the exception of protein derivatives from adenocarcinoma tissue. There was no evidence of statistical interaction between storage duration and cancer tumor tissue type on the concentration of DNA, RNA and miRNA.

### Fit for purpose statistics

#### Fitness of DNA derivatives from FFPE

The distribution of DNA by fitness category was determined by absorbance ratio (A_260_/_280_) and by concentration when stratified by storage duration interval of 11 years and tumor tissue type (Table 2). A Cochran-Mantel-Haenszel (CMH) test showed no significant associations of fitness category with the absorbance values or concentrations of DNA by storage duration in 11 year intervals (*P* = 0.37, *P* = 0.80; respectively), or cancer tumor tissue type (*P* = 0.70, *P* = 0.32; respectively).

#### Fitness of RNA derivatives from FFPE

The distribution of RNA by fitness category was determined by absorbance ratio (A_260_/_280_) and concentration when stratified by storage duration intervals of 11 years and cancer tumor tissue type (Table 3). Storage duration group was associated with RNA fitness based on absorbance values (CMH test, *P = 0.05). No association was observed between cancer tumor tissue type and fitness based on absorbance (CMH test, *P* = 0.14). A Cochran-Mantel-Haenszel (CMH) test showed no significant associations of fitness category with the concentration values of RNA by storage duration in 11 year intervals or cancer tumor tissue type (*P* = 0.25, *P* = 0.13; respectively). The RIN fitness of RNA was somewhat associated with storage duration or cancer tumor tissue type (*P* = 0.08, *P* = 0.07; respectively).

#### Fitness of miRNA derivatives from FFPE

The fitness of miRNA absorbance ratios (A_260_/_280_) were not associated with FFPE storage duration or tumor tissue type (CMH test, *P* = 0.29, *P* = 0.25, respectively) (Table 4). The concentration fitness of miRNA derived from FFPE was not associated with storage duration or tumor tissue type (CMH test, *P* = 0.18, *P* = 0.32; respectively).

#### Fitness of protein derivatives from FFPE

The fitness of proteins derived from FFPE by was not observd to be related to storage duration but was related to tumor tissue type (CMH test, *P* = 0.22, *P = 0.05; respectively) (Table 5).

#### Overall fitness of FFPE blocks

The overall fitness of FFPE blocks, based on the combined assessment of derivatives co-extracted from each block, was not associated with storage duration (CMH test, *P* = 0.26) (Table 6). When analyzed by cancer tissue type, adenocarcinoma FFPE tissue bocks had significantly better overall block fitness than either papillary or squamous FFPE blocks (CMH test, *P = 0.01).

**Table 6:** The fitness from formalin-fixed paraffin-embedded (FFPE) blocks tissue stratified by the storage duration (11 year intervals) and three tumor tissue types (n = 118) as categorized in Table 1.

	1990–2001							
	Bad Block		Specific nucleic acid or protein		Nucleic Acids only		Fit for Diverse Analyses	
Tissue Type	*n*	%	*n*	%	*n*	%	*n*	%
Papillary	2	10	10	50	2	10	6	30
Adenocarcinoma	1	5	2	10	2	10	15	75
Squamous	3	15	6	30	8	40	3	15
Total	6	10	18	30	12	20	24	40
	2002–2013							
	Bad Block		Specific nucleic acid or protein		Nucleic Acids only		Fit for Diverse Analyses	
Tissue Type	*n*	%	*n*	%	*n*	%	*n*	%
Papillary	0	0	8	40	2	10	10	50
Adenocarcinoma	1	6	4	22	4	22	9	50
Squamous	0	0	8	40	4	20	8	40
Total	1	2	20	34	10	17	27	47

#### Dependence of derivative fitness when co-extracted from the same FFPE block

The fitness of one type of derivative extracted from an FFPE block was weakly associated with the fitness of other derivatives co-extracted from the same FFPE block when analyzed by pair-wise Spearman rank correlation matrix (Table 7). In other words, the quality of one derivative in a block did predict the quality of another derivative from the same block.

**Table 7:** Matrix of Spearman correlation coefficients between the numbers of failed derivatives in any set of two molecular extracts from the same block.

Derivative	DNA	RNA	miRNA
RNA	0.48****		
miRNA	0.52****	0.58****	
Protein	0.33***	0.30***	0.16*

****p ≤ 0.0001

***p ≤ 0.001

*p ≤ 0.10

## Discussion

While previous studies have reported on the fitness of individual nucleic acids or protein extracted from FFPE tissue blocks for genomic and proteomic analyses [1, 2, 5–8, 14–20], the current study is unique in its systematic assessment of the fitness of nucleic acids and protein co-extracted from the same FFPE block. A critical tool in the current study was stratified random sampling. While this method is common in population-based studies, it is seldom used for quality assurance studies in biorepositories, despite the fact that biorepositories are particularly amenable to stratified random sampling for two reasons: (1) the attributes of each sampling unit (e.g., the FFPE block) are readily available (e.g., tumor tissue type, duration of storage, etc.) and (2) the numbers of sampling units falling into each strata are known and do not need to be estimated. This sampling method enabled us to stratify the FFPE blocks on two factors that we hypothesized would most affect derivative fitness: (1) storage duration and (2) tumor tissue type. With equal numbers of FFPE blocks per strata (i.e., a “balanced study design”), the study was powered to detect robust differences in FFPE derivatives based on these two critical factors. Moreover, nucleic acids and proteins in FFPE blocks are extremely sensitive to pre-analytical factors, such as the time between tissue acquisition and fixation, tissue autolysis, or temperature during the paraffin embedding process [2]. Unfortunately, pre-analytical factors are seldom annotated for archived FFPE blocks. Morever, durng the 26 years in which these FFPE were archived, numerous developments in formalin fixation methods have been implemented. As the effects of all these pre-analytical factors could not be determined [21], the random stratified sampling used in this study diminished the potential confounding and bias from different pre-analytical factors in the preparation of FFPE tissue.

An important innovation of the current study was to combine the fitness levels of the derivatives coextracted from each FFPE block into a single score for the entire block, which enabled assessment of “overall” block fitness. This led to two important observations in the current study: (1) the fitness of one type of nucleic acid or protein derivative did not predict the fitness of another derivative co-extracted from the same FFPE block; e.g., a block with “unfit” RNA may have “fit” miRNA; and (2) the majority of the FFPE blocks had nucleic acids and protein derivatives “fit” for diverse genomic and proteomic purposes (Table 6). Another finding was that derivatives from adenocarcinoma tissue tended to be more “fit” than derivatives from FFPE containing papillary or squamous cell tumor tissue, an observation which deserves further study.

Some FFPE derivatives survive the extraction and purification better than others. As expected, long-stranded RNA were very labile, especially when RIN was taken into account. Numerous explanations have been proposed to explain the modifications that RNA undergoes as a result of formalin fixation, with the principal change due to the addition of a hydroxymethyl group (methylol) to the nucleic acid backbones, which facilitates the formation of methylene bridges between amino groups within the nucleic acid chain (see [4] for an excellent review of this process). In addition, formaldehyde facilitates depurination (i.e., loss of the purine base while leaving the DNA backbone intact) of RNA as well as hydrolysis of phosphodiester bonds, leading to the short chains of RNA extracted from FFPE tissue (see also [14, 19]). However, it should be emphasized that even though RNA derivatives from FFPE tissue were degraded, which limits their use in many kinds of sequencing analyses, the remaining short strands of RNA remained long enough to be utilized as templates for several genomic methods such as PCR, which uses smaller amplicon sizes (400 base pairs) [18, 22–24]. The fitness of miRNA contrasted clearly with the fitness of co-extracted RNA (Table 3). These are non-coding RNAs of approximately 20–22 nucleotides in length, making them less prone to degradation and modification than total RNA due to their already short length and their close association with large protein aggregates [15, 16, 25, 26]. There are several studies that have directly compared miRNA and RNA fitness from paired FFPE blocks and frozen tissue samples, which demonstrated the better fitness of miRNA compared to RNA [15, 16, 25, 26].

In keeping with numerous recent studies of the quantity and quality of DNA extracted from FFPE tissue [20], we observed DNA derivatives to be easier to extract, more stable, and less prone to degradation than RNA. Finally, FFPE blocks yield potentially amplifiable short amplicons (268 base pairs) for nearly all DNA extracted. Importantly, there was no evidence of a difference in the fitness of DNA after long-term storage or one of the three tumor tissue types studied, which is in agreement with several other studies that suggest that the ability to amplify DNA is not diminished with the aging of FFPE blocks [2, 21, 27, 28].

Finally, more than half of the protein derivatives from FFPE were fit for proteomic purposes. The proteins from adenocarcinoma FFPE tissue were especially more “fit” than proteins from squamous cell or papillary FFPE tissue. A significant interaction effect was observed for the absorbance value for proteins, implying that storage duration affected proteins derivatives from tumor tissue types differently. The mean A_280_ for proteins derived from recently stored FFPE (2002–2013) was greater than mean A_280_ for proteins from older FFPE (1990–2001), with the exception of protein derivatives from adenocarcinoma tissue.

There are several limitations to the current study that need to be addressed. The principle limitation is that the samples were not further stratified by the tumor “site” (or organ), which could be a critical determinant of the fitness of the derivatives extracted from the FFPE. However, if the samples were further stratified by tumor site (e.g., skin, lung, liver, etc.), then the number of FFPE blocks in each sample strata would have been small, lowering the statistical power of study. Two technical issues also limit the current study. While the derivatives often fell unambiguously into the fitness categories of “unfit, “fit”, and “above fit”, which were based on A_260/280_ ratio for nucleic acids and the A_280_ for proteins, a sample with a borderline absorbance (e.g. an miRNA derivative with a A_260/280_ ratio at 1.4996) was difficult to place into a fitness category; i.e., the fitness categories lose their precision along the border ranges. Finally, protein concentration was determined only by A_280_ without the benefit of an extinction coefficient or by more advanced methodologies such as amino acid analysis (AAA) or nitrogen content. While quantifying protein in this study by A_280_ was convenient and does not consume large amounts of material, proteins have widely varying absorption characteristics due to amino acid content. As such, there is some error in this reading, especially as these were protein mixtures: i.e., non-protein components in the solution may also absorb ultraviolet light in addition to the heterogeneous mixture of proteins present. However, the extinction coefficient can only be calculated accurately when the protein sequence is known and then requires further confirmation through other analyses, such amino acid analysis or light scattering detection and refractive index measurement, all of which were beyond the scope this study.

In summary, we present a unique approach for quality control of derivatives from FFPE that differs from previous studies [6–8]) as follows: (a) the fitness of FFPE tissue derivatives can be classified into readily applicable categories for downstream application by bench researchers; (b) multiple types of nucleic acids and protein derivatives can be co-extracted from the same FFPE block, enabling the scoring of overall FFPE block quality; and (c) sampling and statistical methods can be used that enabled reliable estimates of derivative fitness, despite the unknown variation in pre-analytical processing of FFPE blocks. However, much like these previous studies [6–8], the current study shows that FFPE blocks remain “a valuable and underexploited resource” [6] for contemporary genomic and proteomic research.

## Supporting information

S1 File Sizing range (base pair) along with peak (base pair size) of the DNA present in samples. (XLSX)

S2 File SAS code used in this study for ordinal fitness classification. (DOCX)
