# Supplementary material for: Fit for genomic and proteomic purposes: Sampling the fitness of nucleic acid and protein derivatives from formalin fixed paraffin embedded tissue
Source: PLoS One. 2017 Jul 25;12(7):e0181756. doi: 10.1371/journal.pone.0181756 (PMC5526578; doi:10.1371/journal.pone.0181756)
Supplement: S2 File — (DOCX) [file pone.0181756.s002.docx]

**S1 Appendix**. SAS code used in this study for ordinal fitness classification.

*if DNA260_280 < 1.5 or DNA260_280 > 2.5 then D2628cat = 1;*

*else if 1.5 le DNA260_280 le 1.8 or 2.2 le DNA260_280 le 2.5 then D2628cat = 2;*

*else if 1.8 < DNA260_280 < 2.2 then D2628cat = 3;*

*if DNAconc < 5 then DconcCat = 1;*

*else if 5 le DNAconc le 50 then DconcCat = 2;*

*else if DNAconc > 50 then DconcCat = 3;*

*if RNA260_280 < 1.5 or RNA260_280 > 2.5 then R2628cat = 1;*

*else if 1.5 le RNA260_280 le 1.8 or 2.2 le RNA260_280 le 2.5 then R2628cat = 2;*

*else if 1.8 < RNA260_280 < 2.2 then R2628cat = 3;*

*if RNAconc < 5 then RconcCat = 1;*

*else if 5 le RNAconc le 50 then RconcCat = 2;*

*else if RNAconc > 50 then RconcCat = 3;*

*if RIN < 2 then RINcat = 1;*

*else if 2 le RIN le 3 then RINcat = 2;*

*else if RIN > 3 then RINcat = 3;*

*if miRNA260_280 < 1.5 or miRNA260_280 > 2.5 then miR2628cat = 1;*

*else if 1.5 le miRNA260_280 le 1.8 or 2.2 le miRNA260_280 le 2.5 then miR2628cat = 2;*

*else If 1.8 < miRNA260_280 < 2.2 then miR2628cat = 3;*

*if miRNAconc < 5 then miRconcCat = 1;*

*else if 5 le miRNAconc le 50 then miRconcCat = 2;*

*else if miRNAconc > 50 then miRconcCat = 3;*

*if prot_A280 < 1.25 then prot_cat = 1;*

*else if 1.25 le prot_A280 le 2.5 then prot_cat = 2;*

*else if prot_A280 > 2.5 then prot_cat = 3;*
